# Endoscopic Treatment Options in Patients With Gastrojejunal Anastomosis Stricture Following Roux-en-Y Gastric Bypass

**DOI:** 10.4021/gr385w

**Published:** 2012-01-20

**Authors:** Richdeep S. Gill, Kevin A. Whitlock, Rachid Mohamed, Daniel W. Birch, Shahzeer Karmali

**Affiliations:** a Department of Surgery, University of Alberta, Edmonton, Alberta, Canada; b Faculty of Medicine & Dentistry, University of Alberta, Edmonton, Alberta, Canada; c Division of Gastroenterology, University of Calgary, Calgary, Alberta, Canada; d Center of the Advancement of Minimally Invasive Surgery (CAMIS), Royal Alexandria Hospital, Edmonton, Alberta, Canada; e Richdeep S. Gill and Kevin A. Whitlock were co-first authors

**Keywords:** Endoscopy, Gastric bypass, Obesity, Bariatric surgery, Stricture, Balloon dilatation

## Abstract

The proportion of obese individuals continues to increase worldwide. Bariatric surgery remains the only evidence-based treatment strategy to produce marked weight loss. Roux-en-Y gastric bypass is an effective and common bariatric surgical procedure offered to obese patients. However, a small percentage of individuals can develop narrowing or stricture formation of the gastrojejunal anastomosis. Endoscopic treatment of gastrojejunostomy (GJ) is preferred compared to surgical revision, as it is less invasive. The endoscopic treatment strategy most common employed is balloon dilatation. Endoscopic balloon dilatation is successful in majority of cases with low morbidity, however multiple dilatation may be required. Other endoscopic strategies such as incisional therapy has been successful in treating other gastrointestinal anastomotic strictures, however remain to be evaluated in post-RYGB GJ strictures. Further research is needed to determine the effectiveness of incision therapy and other endoscopic treatment strategies compared to endoscopic balloon dilatation.

## Introduction

Roux-en-Y gastric bypass (RYGB) continues to be one of the most common bariatric surgical procedures performed as the obesity epidemic continues to worsen worldwide [1-4]. Currently, bariatric surgery is the only evidence-based approach to achieve sustained and significant weight loss. A RYGB involves the creation of a gastrojejunostomy (GJ), which connects the distal small bowel to the newly created gastric pouch. Stricture formation or scarring of the GJ following RYGB may lead to narrowing and potential obstruction in these patients. The GJ stricture rate following RYGB has been estimated to be 2 - 4% [5-9]. A large prospective study by McCarty et al. reported that 2.1% of their 2000 RYGB patients developed GJ strictures [10]. However, other institutions have reported higher GJ stricture rates, ranging from 5.1 - 6.8% [11-15]. Overall, a GJ stricture rate following RYGB seems to be approximately 2 - 6% with variation among published reports. Treatment of postoperative GJ strictures following RYGB may involve endoscopic management or surgical revision. In this review we will explore endoscopic strategies available to treat GJ strictures in bariatric patients following RYGB.

## Stricture Formation

The underlying etiology of GJ stricture formation is complex and relatively undefined; however numerous factors have been implicated. Though controversial, technical factors may be involved in stricture formation [16]. For example, excessive tension on the GJ anastomosis may promote stricture formation [15]. Furthermore, hand sewn anastomosis, or stapled (linear stapler vs. circular stapler) may affect the stricture rate, with controversy over the superior method [17, 18]. Interestingly, the diameter of the GJ anastomosis is purposely limited provide a restrictive effect [15]. However, a very small diameter may promote stricture formation. Nguyen et al. reported that using a 25 mm circular stapler for the GJ anastomosis was associated with a lower stricture rate when compared to a 21 mm circular stapler [16]. It is suggested that using the 25 mm size stapler provides a 40% increase in cross-sectional area and this leads to decreased rates of stricture formation [16, 19].

Non-technical factors have also been implicated in post-operative stricture formation. Gastric acid from the gastric pouch may cause inflammation and ulceration [15], which may lead to peptic strictures, similar to those seen in the esophagus in patients with gastroesophageal reflux disease [20]. Additonally, Takata et al. propose that ischemia, excessive scar formation, and gastric hypersecretion can all promote stricture formation [21]. Furthermore, smoking and NSAID use are considered modifiable risk factors for gastrointestinal strictures [15, 22, 23]. Therefore, with a better understanding of the etiology of a patient’s GJ stricture, stricture recurrence may be decreased by modifying risk factors.

## Endoscopic Balloon Dilatation

Currently, the most common technique used to treat stricture formation of the gastrojejunal anastomosis is endoscopic balloon dilatation. This technique involves passing an endoscope down the esophagus to the GJ site. Next a “through-the-scope” hydrostatic balloon is positioned across the stoma and inflated under direct visualization. Sizes of inflatable balloons range from 10 mm to 25 mm. Generally, a smaller balloon is chosen for the first dilatation and if that fails then a larger balloon is selected for repeat dilatations. Ultimately, following endoscopic balloon dilatation, the GJ should allow passage of the endoscope to visualize the distal small bowel. Ahmad et al. reported a review of 450 patients who underwent RYGB at their institution [24]. They investigated 14 patients (3.1%) who presented with gastric outlet obstructive symptoms and Upper gastrointestinal (UGI) endoscopy was performed and the GJ was visualized. 13 of these patients had a stricture at the GJ and one patient had marked edema. All patients were treated with endoscopic balloon dilatation; with 64% experiencing symptomatic relief following a single dilatation and 36% requiring further dilatations. This study reported no perforations or complications with endoscopic balloon dilatation.

The most important complication associated with endoscopic balloon dilatation remains perforation. Expansion of the balloon leads to increased transmural pressure, which may result in excessive wall stress, leading to rupture and perforation at the stricture site. Ukleja et al reported a 2.2% incidence of perforation following endoscopic dilatation [13] in a retrospective review of 1012 patients who had undergone RYGB at their institution. Interestingly, endoscopic balloon dilatation was successful following one treatment in only 28% of the patients. The majority (59%) needed two or three dilatations before symptomatic relief. In 2008, Caro et al. specifically investigated the complication rate associated with endoscopic balloon dilatation [25]. They reviewed 111 patients who underwent balloon dilatation following a RYGB, with a total of 200 dilatations performed. They reported one hematoma and two perforations, with all three patients successfully treated conservatively. Based on these findings, Caro et al concluded that endoscopic balloon dilatation is a safe therapy for GJ strictures following RYGB.

The number of dilatations required to successfully treat a gastrojejunal stricture are unknown. Though the risk of perforation is low, repeated endoscopic balloon dilatation has been proposed to increase this risk. A number of reviews have reported that a majority of patients require at least two dilatations to achieve patency of the GJ [13-15]. Goitein et al. reported that only 22% of their patients responded to a single endoscopic balloon dilatation [14]. Their retrospective review also reported a GJ stricture rate of 5.1% based on 369 patients who underwent RYGB surgery. Mathew et al. also concluded that the majority of their patients required more than one dilatation. They retrospectively reviewed 888 patients who underwent RYGB at their institution [15], with 94 patients with obstructive symptoms undergoing radiologic evaluation. Overall, endoscopy diagnosed 58 patients (6.5%) with stricture at the GJ site and an average of 2.2 endoscopic balloon dilatations was performed per patient. Of these pateints, 40% required only one dilatation, with the majority requiring two or more dilatations.

Conversely, other studies have reported successful treatment of GJ strictures in a majority of patients with a single endoscopic balloon dilatation [12, 21, 24]. Peifer et al. retrospective reviewed 801 patients post-RYGB [12] and reported 43 patients (5.4%) with GJ anastomotic strictures. Endoscopic balloon dilatation was performed and successful in 79% of their patients on the first treatment. The authors also reported no perforations or significant bleeding associated with endoscopic therapy. Furthermore a prospective study of 379 morbidly obese patients post-RYGB by Takata et al. also reported 60% of patients requiring only one endoscopic balloon dilatation [21]. These authors also reported no complications following endoscopic balloon dilatation. Thus overall, endoscopic balloon dilatation is an effective treatment strategy in postoperative GJ strictures following RYGB in obese patients. Despite the possibility of multiple dilatations needed to treat the stricture site, endoscopic treatment avoids the need for more invasive surgical revision, which does not necessary reduce the changes of future stricture formation.

## Endoscopic Incisional Therapy

Endoscopic incisional therapy is an alternate technique that has also been employed successfully in the treatment of gastrointestinal strictures. Incisional therapy involves using an energy source to breakdown the scar tissue at the stenotic gastrointestinal site. Schubert et al. reported the use of this alternative therapeutic approach to gastrointestinal strictures [26]. They described the use of argon plasma coagulation with diathermy in 49 patients with anastomotic strictures. Specifically, electroincision was used to created flaps of tissue and the argon plasma coagulation reduced the size of the flaps. Overall this resulted in dilatation of the gastrointestinal lumen by simply ablating the excess tissue. Of note, these patients had colonic or esophageal anastomotic strictures and they had not undergone RYGB. Nonetheless, the technique used by Schubert et al. achieved adequate and long-term recanalization in 92% of patients after a single treatment. Further research is needed to determine if argon plasma coagulation would be useful in RYGB patients with GJ strictures.

Incisional therapy has also been performed with an endoscopic laser. In 2001, Dallal et al. reported the use of endoscopic laser therapy to treat esophageal strictures [27]. Their patients were on palliative treatment for esophageal or esophagogastric carcinoma. All patients were experiencing dysphagia as a result of a diminished patency of their esophagus, 34 patients were randomized into the thermal ablative therapy group (endoscopic laser incision). The majority of cases used either a Nd:YAG laser or an argon diode laser. Recurrent dysphagia was an indicator of success, as dysphagia after the procedure would indicate a failure to resolve the stricture. Dallal et al. found that 79% of their patients did not experience recurrent dysphagia after incisional therapy. However, they also highlighted the limitations of incisional therapy. For instance 6% of their patients experienced esophageal perforations and 9% had tracheoesophageal fistula formation. Furthermore, 21% of their patients required additional laser therapy, balloon dilatation, or placement of a stent.

Consequently, with different endoscopic modalities being used to treat strictures there have been some groups that looked at the effectiveness of combined therapy. In 2003, Suchan et al. reported a prospective review of 94 patients treated endoscopically for colorectal anastomotic strictures [28]. The patients had either malignant pathology or a benign condition in their colorectal area that was surgically treated. This included rectal cancer, colon cancer, diverticular disease, adenoma, and rectal prolapse, among others. The majority of their patients were treated successfully with endoscopic balloon dilatation. However, 30 patients (32%) received a combination of laser or argon plasma coagulation followed by balloon dilatation. The laser or plasma incision was used when the lumen was less than 7 mm. This allowed better passage of the endoscopic balloon when dilatation was performed. Interestingly, 12 patients (13%) were treated successfully with endoscopic laser incision alone and required no balloon dilatation. Suchan et al. also commented that they prefer to use argon plasma coagulation because it as effective as laser incision but does not require as much equipment or safety measures. Therefore, laser or plasma incision likely has a legitimate role in combined therapy for patients with severe stenosis. Again, this study was not specifically looking at GJ strictures, so further research is needed to determine the effectiveness of endoscopic incision techniques in RYGB postoperative care.

## Treating the Etiology of Stricture Formation

Treating the underlying etiology of GJ strictures may be as important as the endoscopic management. Ischemia and gastric acid exposure are thought to play roles in stricture development, and therefore patients should be advised to cease smoking and avoid NSAIDs [15]. Proton pump inhibitors therapy has been beneficial for patients with peptic ulcers or gastric acid hypersecretion [29, 30], and may aid in healing of marginal ulcers to limit GJ stricture formation. Lastly, the technical surgical choices made during the RYGB may decrease stricture formation postoperatively. For example, avoidance of excess tension on the GJ junction may decrease stricture formation [15]. As well, the use of specific materials and methods of securing the junction likely impacts the stricture rate; however the optimal choice is still controversial [8, 15-18]. Appropriate treatment of the underlying risk factors for GJ stricture formation combined with endoscopic management may lead to prevention of GJ stricture formation and reoccurrence.

## Conclusion

RYGB continues to be a popular bariatric surgical procedure as the rates of obesity continue to rise in both North America and throughout the world. Patients effectively achieve weight loss following RYGB, however complications may arise. The GJ site may develop strictures weeks to months following RYGB, leading to obstructive symptoms in the patient. Endoscopy remains a preferred method to treat GJ strictures. Endoscopic balloon dilatation has been shown to be an effective treatment strategy, with a high overall success rate, though multiple dilatations may be required. Endoscopic incisional therapy has been used to successfully treat other GI anastomotic strictures, though its use in specifically treating GJ strictures post-RYGB is limited. Further research is needed to determine the role of incisional therapy in obese patients with postoperative GJ strictures following RYGB.

## References

[1] WHO (2011). Obesity and overweight.

[2] Kelly T, Yang W, Chen CS, Reynolds K, He J (2008). Global burden of obesity in 2005 and projections to 2030. Int J Obes (Lond).

[3] Flegal KM, Carroll MD, Ogden CL, Curtin LR (2010). Prevalence and trends in obesity among US adults, 1999-2008. JAMA.

[4] Tjepkema M (2005). Adult obesity in canada: Measured height and weight. Nutrition: Findings from the Canadian Community Health Survey.

[5] Podnos YD, Jimenez JC, Wilson SE, Stevens CM, Nguyen NT (2003). Complications after laparoscopic gastric bypass: a review of 3464 cases. Arch Surg.

[6] Hwang RF, Swartz DE, Felix EL (2004). Causes of small bowel obstruction after laparoscopic gastric bypass. Surg Endosc.

[7] Blackstone RP, Rivera LA (2007). Predicting stricture in morbidly obese patients undergoing laparoscopic Roux-en-Y gastric bypass: a logistic regression analysis. J Gastrointest Surg.

[8] Nguyen NT, Longoria M, Chalifoux S, Wilson SE (2004). Gastrointestinal hemorrhage after laparoscopic gastric bypass. Obes Surg.

[9] Hanna SC, Jackson C, Rendon S (2010). Laparoscopic Roux-en-Y gastric bypass complicated by a mesocolic jejunal stricture successfully treated with endoscopic TTS balloon dilation. Obes Surg.

[10] McCarty TM, Arnold DT, Lamont JP, Fisher TL, Kuhn JA (2005). Optimizing outcomes in bariatric surgery: outpatient laparoscopic gastric bypass. Ann Surg.

[11] Go MR, Muscarella P, Needleman BJ, Cook CH, Melvin WS (2004). Endoscopic management of stomal stenosis after Roux-en-Y gastric bypass. Surg Endosc.

[12] Peifer KJ, Shiels AJ, Azar R, Rivera RE, Eagon JC, Jonnalagadda S (2007). Successful endoscopic management of gastrojejunal anastomotic strictures after Roux-en-Y gastric bypass. Gastrointest Endosc.

[13] Ukleja A, Afonso BB, Pimentel R, Szomstein S, Rosenthal R (2008). Outcome of endoscopic balloon dilation of strictures after laparoscopic gastric bypass. Surg Endosc.

[14] Goitein D, Papasavas PK, Gagne D, Ahmad S, Caushaj PF (2005). Gastrojejunal strictures following laparoscopic Roux-en-Y gastric bypass for morbid obesity. Surg Endosc.

[15] Mathew A, Veliuona MA, DePalma FJ, Cooney RN (2009). Gastrojejunal stricture after gastric bypass and efficacy of endoscopic intervention. Dig Dis Sci.

[16] Nguyen NT, Stevens CM, Wolfe BM (2003). Incidence and outcome of anastomotic stricture after laparoscopic gastric bypass. J Gastrointest Surg.

[17] Gonzalez R, Lin E, Venkatesh KR, Bowers SP, Smith CD (2003). Gastrojejunostomy during laparoscopic gastric bypass: analysis of 3 techniques. Arch Surg.

[18] Giordano S, Salminen P, Biancari F, Victorzon M (2011). Linear stapler technique may be safer than circular in gastrojejunal anastomosis for laparoscopic Roux-en-Y gastric bypass: a meta-analysis of comparative studies. Obes Surg.

[19] Gould JC, Garren M, Boll V, Starling J (2006). The impact of circular stapler diameter on the incidence of gastrojejunostomy stenosis and weight loss following laparoscopic Roux-en-Y gastric bypass. Surg Endosc.

[20] Richter JE (1999). Peptic strictures of the esophagus. Gastroenterol Clin North Am.

[21] Takata MC, Ciovica R, Cello JP, Posselt AM, Rogers SJ, Campos GM (2007). Predictors, treatment, and outcomes of gastrojejunostomy stricture after gastric bypass for morbid obesity. Obes Surg.

[22] Pohle T, Domschke W (2000). Results of short-and long-term medical treatment of gastroesophageal reflux disease (GERD). Langenbecks Arch Surg.

[23] Trudgill NJ, Smith LF, Kershaw J, Riley SA (1998). Impact of smoking cessation on salivary function in healthy volunteers. Scand J Gastroenterol.

[24] Ahmad J, Martin J, Ikramuddin S, Schauer P, Slivka A (2003). Endoscopic balloon dilation of gastroenteric anastomotic stricture after laparoscopic gastric bypass. Endoscopy.

[25] Caro L, Sanchez C, Rodriguez P, Bosch J (2008). Endoscopic balloon dilation of anastomotic strictures occurring after laparoscopic gastric bypass for morbid obesity. Dig Dis.

[26] Schubert D, Kuhn R, Lippert H, Pross M (2003). Endoscopic treatment of benign gastrointestinal anastomotic strictures using argon plasma coagulation in combination with diathermy. Surg Endosc.

[27] Dallal HJ, Smith GD, Grieve DC, Ghosh S, Penman ID, Palmer KR (2001). A randomized trial of thermal ablative therapy versus expandable metal stents in the palliative treatment of patients with esophageal carcinoma. Gastrointest Endosc.

[28] Suchan KL, Muldner A, Manegold BC (2003). Endoscopic treatment of postoperative colorectal anastomotic strictures. Surg Endosc.

[29] Hershcovici T, Fass R (2011). Pharmacological management of GERD: where does it stand now?. Trends Pharmacol Sci.

[30] Siilin H, Wanders A, Gustavsson S, Sundbom M (2005). The proximal gastric pouch invariably contains acid-producing parietal cells in Roux-en-Y gastric bypass. Obes Surg.

